# Genetic association study identified a 20 kb regulatory element in WLS associated with osteoporosis and bone mineral density in Han Chinese

**DOI:** 10.1038/s41598-017-13932-w

**Published:** 2017-10-20

**Authors:** Dangfeng Zhang, Zhaohui Ge, Xin Ma, Liqiang Zhi, Yunzhi Zhang, Xueyuan Wu, Shuxin Yao, Wei Ma

**Affiliations:** 1 Department of Orthopedics, The First Affiliated Hospital, Xi’an Jiaotong University, Xi’an, Shaanxi China; 2 Department of Joint Surgery, Honghui Hospital, Xi’an Jiaotong University Health Science Center, Xi’an, Shaanxi China; 3 Department of Spine Surgery, General Hospital of Ningxia Medical University, Yinchuan, Ningxia, China; 4 Zhang’s Orthopaedic Hospital, Taizhou, Zhejiang, China; 5 Department of Orthopedics, Shaanxi Provincial People’s Hospital, Xi’an, Shaanxi China

## Abstract

Previous studies have linked the WNT pathway and human skeleton formation; therefore, genes related to WNT might contribute to the onset and development of osteoporosis. In this study, we investigated the potential genetic association of *WLS*, which encodes an important mediator in the WNT pathway, with osteoporosis and its related quantitative traits in a sample of 6,620 individuals from Han Chinese population. A two-stage approach, with a discovery stage with 859 cases and 1,690 controls and a validation stage with 1,039 cases and 3,032 controls, was applied in the study. Forty SNPs were genotyped in the discovery stage. The intronic SNP rs2566752 was identified to be significantly associated with osteoporosis (OR_discovery_ = 0.78, *P*_discovery_ = 3.73 × 10^−5^; OR_validation_ = 0.80, *P*_validation_ = 1.96 × 10^−5^). Two SNPs surrounding rs2566752 (in addition to this SNP itself) were identified to be associated with bone mineral density. In addition, we have identified a 20 kb peak region of H3K27Ac histone mark enrichment between rs2772304 and rs2566752. Our study suggested that *WLS* is an important locus for osteoporosis and its related quantitative phenotypes in Han Chinese population. Additional sequencing-based studies are needed to investigate the genetic architecture of this regulatory region and its relationship with osteoporosis-related phenotypes.

## Introduction

Osteoporosis is a bone-related disorder with the clinical features of decreased bone mineral density (BMD) and increased risk of bone fracture. Age and gender are two of the most correlated risk factors for osteoporosis. The elderly population, especially postmenopausal women, is at high risk for this disorder. The prevalence of osteoporosis was estimated to be 2%–4% worldwide, and it varies slightly in different ethnic groups^1^. Every year, 8.9 million fractures worldwide are due to osteoporosis^2^. It causes a large economic burden for patients and their families^2^. Osteoporosis is a complex disorder to which both environmental and genetic factors contribute^3,4^. Twin studies have determined the heritability of BMD for different bones to be 0.51-0.76^5^. Candidate gene-based association studies have successfully mapped susceptibility for osteoporosis to several loci^6–9^. A meta-analysis conducted by Richards *et al*. has shown that among 150 candidate genes, 9 of them were significantly associated with BMD, although these candidate genes based studies showed very limited consistency^10^. With the development of sequencing technology, genome-wide association study (GWAS) has been considered as a powerful approach for genetic studies of complex diseases^11–17^. So far, more than 40 GWASs focusing on BMD have been published, and 66 total loci have been replicated across all of these studies^18,19^. These susceptibility genes were not randomly distributed across the whole human genome but were clustered around several signalling/metabolism pathways. Estrade *et al*. identified the WNT/β-Catenin pathway, the RANK-RANKL-OPG pathway and the endochondral ossification pathway to be significantly related to BMD through a GWAS-based meta-analysis based on more than 100,000 samples^20^. Early studies have identified that the WNT signalling pathway plays a role in the developmental specification of stem cells and in regeneration^21^. Lim *et al*. discovered that in a genetic model with reduced WNT signalling, osteoprotegerin expression is downregulated, and this molecular disruption results in an increase in osteoclast activity and a decrease in osteoblast activity^22^. This finding linked the WNT pathway and human skeleton formation; therefore, genes related to WNT might contribute to the onset and development of osteoporosis. Among genes related to Wnt signalling pathway, WLS is particularly interesting because of its role played in the regulation of Wnt protein sorting and secretion. Early family based GWAS meta-analysis had identified a significant single nucleotide polymorphism (SNP) within this gene associated with BMD. In this study, we investigated the potential genetic association between *WLS*, which encodes an important mediator in the WNT pathway, and osteoporosis and its related phenotypes using a sample of 6,620 people from the Chinese Han population. A two-stage design was applied for this study, and 40 single nucleotide polymorphisms (SNPs) were genotyped in the discovery stage.

## Methods

### Subjects and measurements of clinical characteristics

A two-stage approach was utilized for single-marker analysis in the study. In the discovery stage, 859 patients with primary osteoporosis and 1,690 age-matched healthy controls were recruited from the First Affiliated Hospital of Xi’an Jiaotong University. In the validation stage, 1,039 patients with primary osteoporosis and 3,032 age-matched healthy controls were enrolled from Taizhou Zhang’s Orthopaedic Hospital. In the study, all individuals were randomly selected and unrelated. Demographic data, including age and body mass index (BMI), were collected (Table 1). The bone mineral density (BMD) of all subjects was measured using dual-energy X-ray absorptiometry (Lunar Expert 1313, Lunar Corp., USA) at the lumbar spine (L2–4) and femoral neck by two qualified radiologists who were blinded to other clinical data. Osteoporosis was diagnosed according to the criteria of the World Health Organization. Subjects with T score <−1.0 SD were grouped into patients as having low bone mass, and those with T score >−1.0 SD were classified into controls. The selected patients with primary osteoporosis met the following criteria. First, they did not have a history or evidence of rheumatoid arthritis, organ transplantation, metabolic bone disease (e.g. hyperthyroidism; hyperparathyroidism; diabetes; Cushing’s disease; liver or kidney dysfunction), or drug treatments including anti-osteoporosis drugs, vitamin D, or others known to affect bone metabolism. Second, they had not had previous fracture or replacement of both hips, any serious chronic liver disease, severe kidney disease, active rheumatologic disease, serious chronic gastrointestinal system disease, history of malignancies, serious cardiovascular disease, or the presence of a prolonged immobility condition (e.g., spinal cord injury, Parkinson’s disease, stroke). None of the subjects had a history of taking medicines for the treatment of osteoporosis or a long history of smoking or alcohol consumption, and subjects with diseases or medications known to affect bone metabolism were excluded from the study. The areal BMD was expressed in grams per square centimetre (g/cm^2^), and the BMD of the lumbar spine and femoral neck are summarized in Table 1. To restrict the genetic background of our study subjects, we excluded anyone who was not born locally, or whose immediate family members from the previous three generations were not born locally. In addition, osteoprotegerin (OPG) levels and soluble receptor activator of nuclear factor-JB ligand (sRANKL) levels of all subjects were measured using enzyme-linked immunosorbent assay (ELISA) kits (Westtang Biotech Inc., Shanghai, China), and the results are presented in Table 1. Written informed consent was obtained from all subjects. This study was performed in accordance with the ethical guidelines of the Declaration of Helsinki (version 2002) and was approved by the Medical Ethics Committee of Xi’an Jiaotong University.

**Table 1:** Demographic characteristics of study subjects.

	Discovery (N = 2,549)		Replication (N = 4,071)	
	Case	Control	Case	Control
Subjects	859	1,690	1,039	3,032
Female/Male	578/281	1134/556	608/431	1780/1252
Age, years (sd)	61.54 (8.08)	61.18 (7.73)	62.32 (6.57)	62.21 (6.64)
BMI, kg/m^2^ (sd)	23.87 (1.50)	23.62 (1.41)	24.11 (1.43)	23.84 (1.34)
LS-BMD, g/cm^2^ (sd)	0.763 (0.07)	0.931 (0.06)	0.866 (0.10)	0.942 (0.07)
FN-BMD, g/cm^2^ (sd)	0.643 (0.07)	0.852 (0.05)	0.772 (0.12)	0.872 (0.06)
OPG, pg/ml (sd)	130.07 (25.42)	178.31 (27.68)	158.82 (35.31)	175.23 (26.71)
sRANKL, pg/ml (sd)	14.76 (4.22)	11.40 (2.52)	12.71 (3.65)	11.49 (2.75)

BMI, body mass index; BMD, bone mineral density; LS, lumbar spine; FN, femoral neck; OPG, osteoprotegerin; sRANKL, soluble receptor activator of nuclear factor-JB ligand.

### SNP selection and genotyping

*WLS* tagging SNPs (tagSNPs) were selected from the 1000 Genomes Chinese Han Beijing population (CHB) using Haploview^23^. SNPs with minor allele frequencies (MAF) ≥0.05 were selected based on pairwise tagging with an *r* ^2^ threshold of 0.5. A final set of 40 tag SNPs was selected within the *WLS* gene (Supplemental Table S1). Genomic DNA was extracted from peripheral blood leukocytes according to the manufacturer’s protocol (Genomic DNA kit, Axygen Scientific Inc., California, USA). Genotyping was performed for all SNPs using the Sequenom Mass ARRAY RS1000 system (Sequenom, San Diego, California, USA). The results were processed using Typer Analyzer software (Sequenom), and genotype data were generated from the samples^24^.

### Power Analyses

We performed power analyses using the Genetic Association Study (GAS) Power Calculator (http://csg.sph.umich.edu/abecasis/CaTS/gas_power_calculator/index.html) for both the discovery stage and the validation stage. For the parameter settings, the prevalence of osteoporosis was approximately 3%, and we assumed that the MAF of the underlying disease marker matched with our candidate SNPs. Then, we used the average MAF of our 40 candidate SNPs as the disease allele frequency (0.23). For the discovery stage, our study subjects comprised 859 cases and 1,690 controls, and we chose α = 0.05 as the significance level. For the validation stage, our sample included 1,039 cases and 3,032 controls. We assumed 5 SNPs would be tested in the validation stage, so we chose α = 0.01 as the significance level in this stage. The results are summarized in Supplemental Table S2. The power analyses showed that our sample size could provide enough power to detect moderate genetic effects (RR >1.2).

### Statistical Analyses

We conducted genetic association analyses to investigate the potential association between osteoporosis and genetic markers on *WLS*. Logistic models were fitted for each genetic marker. Age, Gender, and BMI of study subjects were included in each model as covariates to control for potential confounding effects. A two-stage study design was applied to maximize the cost efficiency of our study. In the discovery stage, we tested 40 SNPs in 2,549 study subjects. Genetic markers with at least marginal significance (*P* < 0.05), and the SNPs in LD with them (D′ > 0.9), were included in the validation stage (second stage), which comprised 4,071 study subjects in total. In addition to the disease status of osteoporosis, we also tested the potential associations between our candidate SNPs and some quantitative traits related to osteoporosis, including the BMD of the femoral neck and lumbar spine (FN-BMD, LS-BMD), and blood levels of OPG and sRANKL. Linear models were fitted to perform the association analyses. Covariates included in the logistic models were also added in the linear models. Considering that analyses of some SNPs are difficult to obtain sufficient data to draw a convincing conclusion^25–27^, we have also conducted haplotype-based analyses to investigate the potential association signals contributed by a combination of multiple genetic markers. Hardy-Weinberg equilibrium test (HWE), MAF calculations, linkage disequilibrium calculations and single marker-based association analyses were conducted by the genetic association software, Plink v1.9^28^. LD block construction and haplotype analyses were performed using Haploview v4.2^23^. Bonferroni correction was applied when necessary to address multiple-comparison problems in both stages. The significant *P*-value thresholds were defined by 0.00125 (0.05/40, the number of SNPs tested) in the discovery stage and 0.008 (0.05/6, the number of SNPs tested) in the validation stage.

### Bioinformatics Analyses

To examine the potential biological significance of the identified SNPs, we analysed levels of enrichment of the H3K27Ac histone mark (from 7 cell lines including Hsmm, Nhlf, Gm12878, Nhek, H1hesc, K562 and Huvec) within the gene region ± 10 kb of *WLS* as determined by a ChIP-seq assay generated by the ENCODE project^29^ through the public bioinformatics analysis platform, Galaxy (https://usegalaxy.org/)^30^, and plotted the distribution of the ChIP-seq signal strength across the genomic region using the ggplot2 package of R project (http://ggplot2.org/). The H3K27Ac histone mark is the acetylation of lysine 27 of the H3 histone protein, and it is often found near active regulatory elements. For a genomic region with high levels of the H3K27Ac histone mark, genetic changes might have significant effects on the transcription of genes and other downstream processes of the central dogma, thus modifying phenotypes. In addition, we determined the potential functional significance of the identified SNPs by RegulomeDB (http://www.regulomedb.org/)^31^.

## Results

All the SNPs were in HWE, with a MAF greater than 0.05 (Supplemental Table S1). For the single marker-based study focusing on the disease status of osteoporosis (qualitative trait), we identified two nominal significant SNPs, rs2772304 (OR = 1.15, *P* = 0.0434) and rs2566752 (OR = 0.78, *P* = 3.73 × 10^−5^) after accounting for age, gender and BMI. In total, six SNPs (two nominal significant SNPs, and another four SNPs with D′ > 0.7 with each of the two SNPs) were tested in the validation stage (the significance level was 0.05/6 = 0.008). The *r* ^2^ and D’ were calculated between the two nominal significant SNPs and all the other SNPs genotyped in discovery stage, and the results were summarized and shown in Supplemental Tables S3 and S4. Only one SNP, rs2566752 (OR = 0.80, *P* = 1.96 × 10^−5^), remained significant after multiple-comparison correction. The results are summarized in Table 2. We constructed 7 LD blocks using data from the discovery stage based on 40 candidate SNPs. Haplotype analyses were performed, and no LD blocks were identified to be significant (Supplemental Table S5).

**Table 2:** Single marker-based association analyses for the 6 SNPs included in the validation stage with respect to osteoporosis.

CHR	SNP	POS	A1	OR_1^*^	STAT_1^*^	*P*_1^*^	OR_2^**^	STAT_2^**^	*P*_2^**^
1	rs2772304	68636580	A	1.15	2.02	0.0434	1.11	1.80	0.0727
1	rs12725769	68637158	C	0.98	−0.29	0.7746	0.99	−0.22	0.8250
1	rs59393705	68644066	C	1.01	0.07	0.9422	0.99	−0.23	0.8183
1	rs2772297	68652084	T	1.04	0.54	0.5895	1.02	0.29	0.7754
1	rs2026749	68653489	G	0.99	−0.22	0.8234	0.99	−0.13	0.8970
1	rs2566752	68656697	C	0.78	−4.12	3.73E-05	0.80	−4.27	1.96E-05

^*^odds ratio, t-statistics, and p values for discovery stage.

^**^odds ratio, t-statistics, and p values for validation stage.

We combined samples from both stages to perform association analyses for the 6 SNPs genotyped during the validation stage, based on quantitative traits (Table 3 and Supplemental Table S6). These results did not align perfectly compared to the results based on disease status. All 6 of the SNPs showed significance in more than one of the quantitative traits. To examine whether these SNPs have independent effects, we fitted linear models conditioned on rs2566752, which is the most significant SNP. The results showed that most of these SNPs maintained their significance in the condition models, and this indicated that all of the significant SNPs might have independent effects. We also conducted association analyses for the discovery-stage samples using quantitative traits, and the results are shown in Supplemental Table S7.

**Table 3:** Results of single marker-based linear regression analyses for femoral neck and lumbar spine.

CHR	SNP	POSITION	A1	BETA_FNBMD	*P*_FNBMD	*P*_FNBMD_Con^*^	BETA_LSBMD	*P*_LSBMD	*P*_LSBMD_Con^*^
1	rs2772304	68636580	A	0.006	0.0072	8.94 × 10^−6^	0.008	8.42 × 10^−5^	0.0002
1	rs12725769	68637158	C	0.014	1.28 × 10^−9^	0.2113	0.014	1.12 × 10^−11^	0.2485
1	rs59393705	68644066	C	0.028	<1 × 10^−16^	9.15 × 10^−6^	0.029	<1 × 10^−16^	0.0010
1	rs2772297	68652084	T	0.031	<1 × 10^−16^	8.89 × 10^−9^	0.032	<1 × 10^−16^	6.53 × 10^−6^
1	rs2026749	68653489	G	0.041	<1 × 10^−16^	<1 × 10^−16^	0.040	<1 × 10^−16^	<1 × 10^−16^
1	rs2566752	68656697	C	0.054	<1 × 10^−16^	—	0.051	<1 × 10^−16^	—

^*^ *P* values of linear regression analyses conditioned on rs2566752.

**Figure 1:**
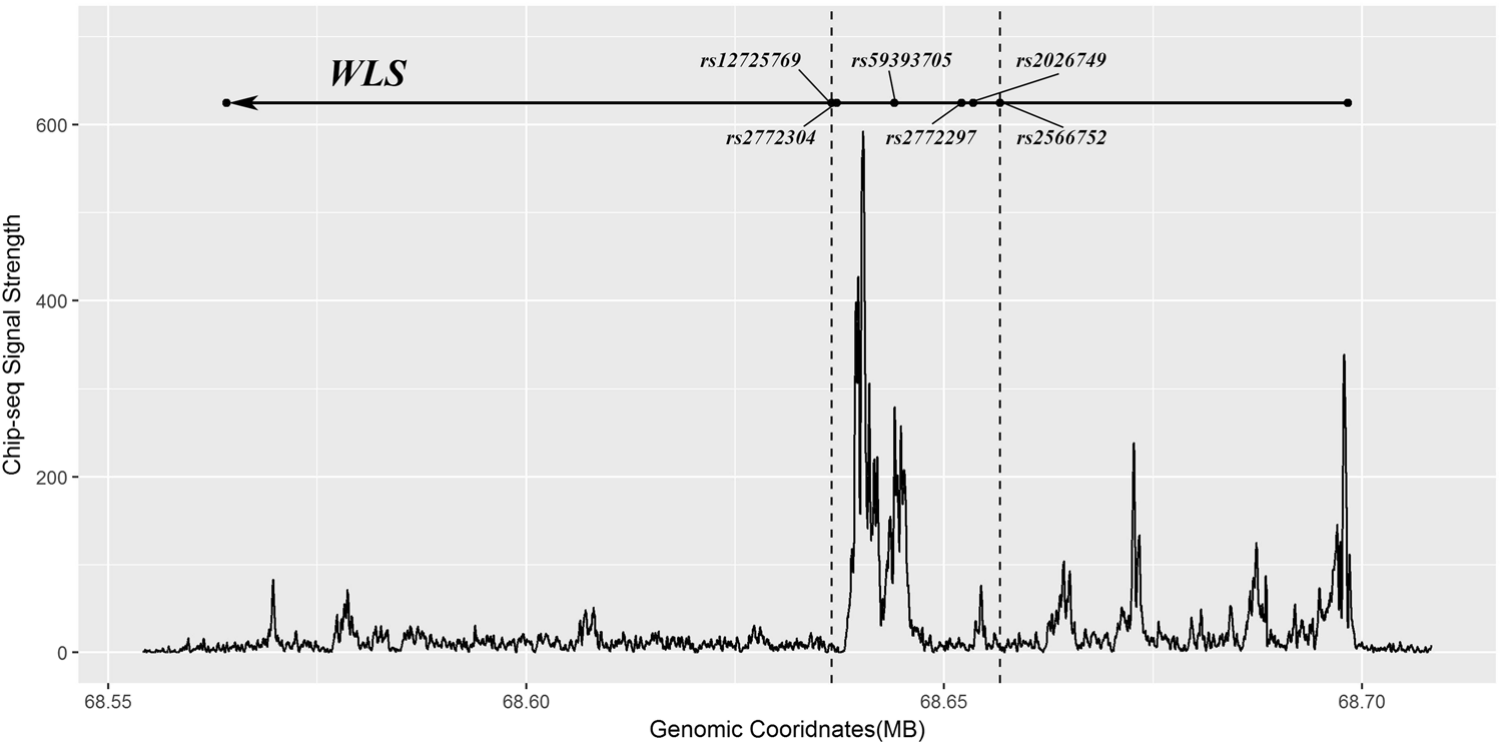
Enrichment of the H3K27Ac histone mark in the gene region of *WLS*.

We investigated the potential functional significance of all 6 SNPs included in the validation stage. Our findings suggest that these SNPs might have very limited functional significance (Supplemental Table S8). RegulomeDB utilizes a score system to evaluate the potential functional effects of SNPs (from 0-6). A low RegulomeDB score indicates that a SNP is more likely to be functional. As we can see from the Table, most of the 6 SNPs have a score of 5 or 6, which means that they have very limited functional significance, while the most significant SNP, rs2566752, has no score to show at all. In addition, we plotted the levels of enrichment of the H3K27Ac histone mark (from 7 cell lines), as determined by ChIP-seq assay using ENCODE data, within the gene region ± 10 kb of *WLS* (Fig. 1). As we can see from Fig. 1, we identified a 20 kb peak region for the H3K27Ac histone mark between rs2772304 and rs2566752. This peak region perfectly overlapped our significant findings in the association analyses using quantitative traits.

## Discussion

*WLS* (Wntless Wnt Ligand Secretion Mediator) is a protein-coding gene that encodes a regulator of Wnt protein sorting and secretion in a feedback regulatory mechanism. Our study has identified a SNP, rs2566752, to be significantly associated with the disease status of osteoporosis in a large sample from the Chinese Han population. Further analyses based on quantitative traits related to osteoporosis have extended our significant findings from a single SNP to a 20 kb genomic region by integrating ENCODE H3K27Ac histone mark data. A previous GWAS meta-analysis had identified rs2566752 to be significantly associated with BMD^32^. In that study, Mullin *et al*. combined two family cohorts and conducted a family-based design to investigate potential genetic effects on BMD^32^. At least two potential limitations might hinder the results of this previous study. The first one is that the previous study was based on family samples, which is not popular in GWAS because of the difficulty of addressing the familial correlations. Even if GEMMA can remove the familial relatedness completely, it might hinder the accuracy of the study because of its conservativeness. In addition, in Mullin’s study, the proportion of male subjects is very low (<10%), which will create a representation problem. For our study, the numbers of males and females were comparable. Above all, in this sense, the results of our study of the Chinese Han population are more than a replication of the previous GWAS-based meta-analysis. The results of our study might offer a more accurate and comprehensive estimation for the genetic effects of rs2566752 on BMD.

H3K27 is known for its effects on downregulating transcription. When H3K27 is trimethylated (H3K27me3), it is tightly associated with inactive gene promoters. On the other hand, acetylation of H3K27 is expected to be antagonistic to the repression of gene transcription/expression by H3K27me3. The bioinformatics analyses integrated with ENCODE data have identified a 20 kb H3K27Ac enrichment region in an intron of *WLS*. In this region, we have identified 6 SNPs to be significantly associated with BMD, and the results of conditional linear regressions indicated that the effects of the other 5 SNPs were independent of rs2566752. However, the bioinformatics analyses did not support the functional significance of all of these 6 SNPs. A possible explanation for this contradiction is that we might have missed the real underlying causative variants in this study, and all of these 6 significant SNPs (including the most significant one, rs2566752) were just surrogates of these underlying variants with true effects. It is probable that multiple ungenotyped genetic variants, including both common polymorphisms and rare/low-frequency variants within this 20 kb region, contributed to the susceptibility of subjects to osteoporosis and BMD. It is impossible to scrutinize this hypothesis from the scope of this study and the previous, low-density common polymorphism-based GWAS study. In the future, targeted deep sequencing or genome-wide sequence studies are needed to unravel the genetic architecture of this regulatory region related to BMD and osteoporosis.

Despite the advantages we discussed above, our study also suffered from several limitations. Population stratification is one of the major threats to the credibility of our significant findings. In a candidate gene-based study that only included 40 SNPs, it is unrealistic for us to perform some methods regularly used in GWAS, such as principle component analysis (PCA). Nevertheless, we have tried our best to remove the potential effects of population stratification by restricting the genetic background (through confining the immigration history of our study subjects) during the sample collection process^33,34^. Combined with the results of previous studies, our significant findings of rs2566752 and its surrounding SNPs have little chance to be a false-positive signal. Another disadvantage of our study is that we only genotyped a very small set of SNPs. As we have discussed above, our findings have offered a clue to the potential role of a 20 kb regulatory genomic region in osteoporosis and BMD. However, we only genotyped 6 SNPs within this region, which is not nearly enough to clearly depict the whole picture of the genetic architecture of this genomic region. Considering the limitation of experimental cost in our study, the only thing we can do to extend our genetic marker coverage is to conduct imputation analyses. However, the accuracy and reliability of imputations are doubtful. In some previous work using the imputation technique, the results were difficult to explain and only generated more confusion and some supplemental tables and figures. Therefore, in this study, we did not conduct imputation analyses. We believe that although our findings might be incomplete in some sense, they are still informative and valuable for the issues we focused on in this study.

## Conclusions

To sum up, in this study, we have shown that *WLS* is an important locus for osteoporosis and its related quantitative phenotypes in a large sample derived from the Chinese Han population. In addition to the significant single SNPs that we identified, a possible regulatory role for the 20 kb genomic region around the most significant SNP, rs2566752, was defined by combining our results with ENCODE histone mark data. Sequencing-based studies are needed in the future to investigate the genetic architecture of this regulatory region and its relationship with osteoporosis-related phenotypes.

## Electronic supplementary material

Supplementary Information
