# Supplementary material for: Genetic association study identified a 20 kb regulatory element in WLS associated with osteoporosis and bone mineral density in Han Chinese
Source: Sci Rep. 2017 Oct 20;7:13668. doi: 10.1038/s41598-017-13932-w (PMC5651806; doi:10.1038/s41598-017-13932-w)
Supplement: Supplementary file 1 — Supplementary Information [file 41598_2017_13932_MOESM1_ESM.doc]

***Title***: Genetic association study identified a 20kb regulatory element in WLS associated with osteoporosis and bone mineral density in Han Chinese

***Author names and affiliations***: Dangfeng Zhang ^a^, Zhaohui Ge ^c^, Xin Ma ^a^, Liqiang Zhi ^b^, Yunzhi Zhang ^d^, Xueyuan Wu ^e^, Shuxin Yao ^b^ and Wei Ma ^a^

^a^ Department of Orthopedics, the First Affiliated Hospital, Xi'an Jiaotong University, Xi’an, Shaanxi, China;

^b^ Department of Joint Surgery, Honghui Hospital, Xi’an Jiaotong University Health Science Center, Xi’an, Shaanxi, China;

^c^ Department of Spine Surgery, General Hospital of Ningxia Medical University，Yinchuan, Ningxia, China;

^d^ Zhang’s Orthopaedic Hospital, Taizhou, Zhejiang, China;

^e^ Department of Orthopedics, Shaanxi Provincial People's Hospital, Xi'an, Shaanxi, China.

***Corresponding Author***:

Shuxin Yao, M.D. & Ph.D., Department of Joint Surgery, Honghui Hospital, Xi’an Jiaotong University Health Science Center, No.555, Youyi East Road, Xi'an, Shaanxi, China, 710054.

Tel: 86-29-88418009; Fax: 86-29-62818386; E-mail: popgold@163.com

Wei Ma, M.D. & Ph.D., Department of Orthopedic, the First Affiliated Hospital of Xi'an Jiaotong University, No.277, Yanta West Road, Xi'an, Shaanxi, China, 710061.

Tel: 86-29-85323935; Fax: 86-029-85323935; E-mail: mawei60@126.com

Supplemental Table S1. Information of the 40 selected SNPs in discovery stage.

| CHR | SNP | POS | ALLELE | FUNC | GENE | MAF | HWE |
| --- | --- | --- | --- | --- | --- | --- | --- |
| 1 | rs1033973 | 68566993 | C/T | intron | *WLS* | 0.15 | 0.85 |
| 1 | rs11209214 | 68567299 | C/T | intron | *WLS* | 0.09 | 0.89 |
| 1 | rs9436797 | 68567418 | A/G | intron | *WLS* | 0.09 | 0.78 |
| 1 | rs11580462 | 68567441 | A/G | intron | *WLS* | 0.27 | 0.95 |
| 1 | rs1033974 | 68567812 | C/G | intron | *WLS* | 0.22 | 0.94 |
| 1 | rs1033975 | 68567848 | A/G | intron | *WLS* | 0.17 | 0.86 |
| 1 | rs11805150 | 68568921 | C/T | intron | *WLS* | 0.10 | 0.79 |
| 1 | rs6671127 | 68571220 | A/C | intron | *WLS* | 0.34 | 0.87 |
| 1 | rs12738528 | 68576340 | C/G | intron | *WLS* | 0.07 | 0.73 |
| 1 | rs187922 | 68576820 | A/G | intron | *WLS* | 0.37 | 0.80 |
| 1 | rs76551413 | 68581841 | A/G | intron | *WLS* | 0.09 | 0.45 |
| 1 | rs12741473 | 68581999 | A/C | intron | *WLS* | 0.19 | 0.94 |
| 1 | rs2270 | 68585783 | C/T | intron | *WLS* | 0.10 | 0.79 |
| 1 | rs2820502 | 68586404 | C/T | intron | *WLS* | 0.07 | 0.29 |
| 1 | rs1046852 | 68591104 | C/T | intron | *WLS* | 0.40 | 0.84 |
| 1 | rs7062 | 68591253 | A/G | intron | *WLS* | 0.44 | 0.73 |
| 1 | rs56149204 | 68594560 | A/G | intron | *WLS* | 0.28 | 0.95 |
| 1 | rs145687155 | 68601508 | C/T | intron | *WLS* | 0.07 | 0.69 |
| 1 | rs2052958 | 68609355 | C/G | intron | *WLS* | 0.18 | 0.87 |
| 1 | rs76918505 | 68623898 | A/G | intron | *WLS* | 0.08 | 0.86 |
| 1 | rs1367448 | 68633924 | C/T | intron | *WLS* | 0.47 | 1.00 |
| 1 | rs1367447 | 68634030 | A/G | intron | *WLS* | 0.22 | 0.94 |
| 1 | rs2772304 | 68636580 | G/T | intron | *WLS* | 0.24 | 0.68 |
| 1 | rs12725769 | 68637158 | A/C | intron | *WLS* | 0.24 | 0.84 |
| 1 | rs59393705 | 68644066 | A/C | intron | *WLS* | 0.25 | 0.79 |
| 1 | rs2772297 | 68652084 | A/G | intron | *WLS* | 0.22 | 0.94 |
| 1 | rs2026749 | 68653489 | A/C | intron | *WLS* | 0.46 | 0.96 |
| 1 | rs2566752 | 68656697 | A/G | intron | *WLS* | 0.48 | 1.00 |
| 1 | rs2116047 | 68661751 | C/T | intron | *WLS* | 0.43 | 0.92 |
| 1 | rs75437229 | 68662739 | A/T | intron | *WLS* | 0.08 | 0.64 |
| 1 | rs7554551 | 68665023 | C/T | intron | *WLS* | 0.21 | 0.89 |
| 1 | rs7511845 | 68665174 | C/T | intron | *WLS* | 0.32 | 0.96 |
| 1 | rs2820503 | 68666724 | A/G | intron | *WLS* | 0.27 | 0.85 |
| 1 | rs17130583 | 68674957 | C/G | intron | *WLS* | 0.24 | 0.95 |
| 1 | rs4655788 | 68677550 | C/G | intron | *WLS* | 0.15 | 0.92 |
| 1 | rs12034030 | 68677760 | C/T | intron | *WLS* | 0.23 | 0.89 |
| 1 | rs76039723 | 68680366 | C/T | intron | *WLS* | 0.13 | 1.00 |
| 1 | rs989842 | 68680929 | A/G | intron | *WLS* | 0.08 | 0.74 |
| 1 | rs4655789 | 68694251 | G/T | intron | *WLS* | 0.33 | 1.00 |
| 1 | rs79249533 | 68695408 | A/T | intron | *WLS* | 0.45 | 0.88 |

Supplemental Table S2. Results of the power analyses.

| Discovery Stage (α=0.05) | | Validation Stage(α=0.01) | |
| --- | --- | --- | --- |
| Genotype Relative Risk | Power | Genotype Relative Risk | Power |
| 1 | 0.05 | 1 | 0.01 |
| 1.1 | 0.28 | 1.1 | 0.17 |
| 1.15 | 0.52 | 1.15 | 0.40 |
| 1.2 | 0.74 | 1.2 | 0.67 |
| 1.25 | 0.89 | 1.25 | 0.87 |
| 1.3 | 0.96 | 1.3 | 0.96 |
| 1.35 | 0.99 | 1.35 | 0.99 |
| 1.4 | 1.00 | 1.4 | 1.00 |
| 1.45 | 1.00 | 1.45 | 1.00 |
| 1.5 | 1.00 | 1.5 | 1.00 |

Supplemental table S3. r^2^ and D’ between rs2566752 and other SNPs genotyped in the discovery stage.

| CHR_A | SNP_A | CHR_B | SNP_B | r^2^ | D’ |
| --- | --- | --- | --- | --- | --- |
| 1 | rs2566752 | 1 | rs1033973 | 0.058 | 0.551 |
| 1 | rs2566752 | 1 | rs11209214 | 0.098 | 0.326 |
| 1 | rs2566752 | 1 | rs9436797 | 0.083 | 0.300 |
| 1 | rs2566752 | 1 | rs11580462 | 0.051 | 0.357 |
| 1 | rs2566752 | 1 | rs1033974 | 0.032 | 0.186 |
| 1 | rs2566752 | 1 | rs1033975 | 0.022 | 0.154 |
| 1 | rs2566752 | 1 | rs11805150 | 0.043 | 0.598 |
| 1 | rs2566752 | 1 | rs6671127 | 0.081 | 0.296 |
| 1 | rs2566752 | 1 | rs12738528 | 0.098 | 0.326 |
| 1 | rs2566752 | 1 | rs187922 | 0.006 | 0.097 |
| 1 | rs2566752 | 1 | rs76551413 | 0.009 | 0.099 |
| 1 | rs2566752 | 1 | rs12741473 | 0.08 | 0.294 |
| 1 | rs2566752 | 1 | rs2270 | 0.069 | 0.757 |
| 1 | rs2566752 | 1 | rs2820502 | 0.068 | 0.271 |
| 1 | rs2566752 | 1 | rs1046852 | 0.097 | 0.324 |
| 1 | rs2566752 | 1 | rs7062 | 0.046 | 0.232 |
| 1 | rs2566752 | 1 | rs56149204 | 0.127 | 0.371 |
| 1 | rs2566752 | 1 | rs145687155 | 0.037 | 0.200 |
| 1 | rs2566752 | 1 | rs2052958 | 0.000 | 0.036 |
| 1 | rs2566752 | 1 | rs76918505 | 0.000 | 0.076 |
| 1 | rs2566752 | 1 | rs1367448 | 0.011 | 0.106 |
| 1 | rs2566752 | 1 | rs1367447 | 0.012 | 0.196 |
| 1 | rs2566752 | 1 | rs2772304 | 0.051 | 0.380 |
| 1 | rs2566752 | 1 | rs12725769 | 0.063 | 0.422 |
| 1 | rs2566752 | 1 | rs59393705 | 0.215 | 0.773 |
| 1 | rs2566752 | 1 | rs2772297 | 0.259 | 0.926 |
| 1 | rs2566752 | 1 | rs2026749 | 0.839 | 0.952 |
| 1 | rs2566752 | 1 | rs2566752 | 1.000 | 1.000 |
| 1 | rs2566752 | 1 | rs2116047 | 0.101 | 0.352 |
| 1 | rs2566752 | 1 | rs75437229 | 0.010 | 0.323 |
| 1 | rs2566752 | 1 | rs7554551 | 0.001 | 0.050 |
| 1 | rs2566752 | 1 | rs7511845 | 0.002 | 0.055 |
| 1 | rs2566752 | 1 | rs2820503 | 0.000 | 0.003 |
| 1 | rs2566752 | 1 | rs17130583 | 0.000 | 0.029 |
| 1 | rs2566752 | 1 | rs4655788 | 0.000 | 0.007 |
| 1 | rs2566752 | 1 | rs12034030 | 0.000 | 0.000 |
| 1 | rs2566752 | 1 | rs76039723 | 0.000 | 0.031 |
| 1 | rs2566752 | 1 | rs989842 | 0.024 | 0.161 |
| 1 | rs2566752 | 1 | rs4655789 | 0.014 | 0.123 |
| 1 | rs2566752 | 1 | rs79249533 | 0.012 | 0.116 |

Supplemental table S4. r^2^ and D’ between rs2772304 and other SNPs genotyped in the discovery stage.

| CHR_A | SNP_A | CHR_B | SNP_B | r^2^ | D’ |
| --- | --- | --- | --- | --- | --- |
| 1 | rs2772304 | 1 | rs1033973 | 0.038 | 0.261 |
| 1 | rs2772304 | 1 | rs11209214 | 0.004 | 0.113 |
| 1 | rs2772304 | 1 | rs9436797 | 0.045 | 0.379 |
| 1 | rs2772304 | 1 | rs11580462 | 0.021 | 0.157 |
| 1 | rs2772304 | 1 | rs1033974 | 0.091 | 0.319 |
| 1 | rs2772304 | 1 | rs1033975 | 0.012 | 0.136 |
| 1 | rs2772304 | 1 | rs11805150 | 0.052 | 0.384 |
| 1 | rs2772304 | 1 | rs6671127 | 0.006 | 0.099 |
| 1 | rs2772304 | 1 | rs12738528 | 0.001 | 0.065 |
| 1 | rs2772304 | 1 | rs187922 | 0.002 | 0.061 |
| 1 | rs2772304 | 1 | rs76551413 | 0.066 | 0.459 |
| 1 | rs2772304 | 1 | rs12741473 | 0.002 | 0.052 |
| 1 | rs2772304 | 1 | rs2270 | 0.032 | 0.302 |
| 1 | rs2772304 | 1 | rs2820502 | 0.000 | 0.010 |
| 1 | rs2772304 | 1 | rs1046852 | 0.001 | 0.051 |
| 1 | rs2772304 | 1 | rs7062 | 0.001 | 0.046 |
| 1 | rs2772304 | 1 | rs56149204 | 0.001 | 0.078 |
| 1 | rs2772304 | 1 | rs145687155 | 0.000 | 0.110 |
| 1 | rs2772304 | 1 | rs2052958 | 0.000 | 0.013 |
| 1 | rs2772304 | 1 | rs76918505 | 0.006 | 0.151 |
| 1 | rs2772304 | 1 | rs1367448 | 0.071 | 0.447 |
| 1 | rs2772304 | 1 | rs1367447 | 0.177 | 0.443 |
| 1 | rs2772304 | 1 | rs2772304 | 1.000 | 1.000 |
| 1 | rs2772304 | 1 | rs12725769 | 0.879 | 0.940 |
| 1 | rs2772304 | 1 | rs59393705 | 0.196 | 0.448 |
| 1 | rs2772304 | 1 | rs2772297 | 0.153 | 0.422 |
| 1 | rs2772304 | 1 | rs2026749 | 0.054 | 0.375 |
| 1 | rs2772304 | 1 | rs2566752 | 0.051 | 0.380 |
| 1 | rs2772304 | 1 | rs2116047 | 0.009 | 0.141 |
| 1 | rs2772304 | 1 | rs75437229 | 0.001 | 0.051 |
| 1 | rs2772304 | 1 | rs7554551 | 0.000 | 0.006 |
| 1 | rs2772304 | 1 | rs7511845 | 0.000 | 0.022 |
| 1 | rs2772304 | 1 | rs2820503 | 0.043 | 0.224 |
| 1 | rs2772304 | 1 | rs17130583 | 0.04 | 0.200 |
| 1 | rs2772304 | 1 | rs4655788 | 0.018 | 0.179 |
| 1 | rs2772304 | 1 | rs12034030 | 0.031 | 0.181 |
| 1 | rs2772304 | 1 | rs76039723 | 0.045 | 0.308 |
| 1 | rs2772304 | 1 | rs989842 | 0.045 | 0.404 |

Supplemental Table S5. Results of the haplotype analyses.

| SNPS | Alleles | Haplotype Freq. | Case/Control Freq. | STAT | *P* |
| --- | --- | --- | --- | --- | --- |
| rs11580462|rs1033974 |  |  |  |  |  |
|  | AG | 0.729 | 0.729/0.729 | 0.001 | 0.979 |
|  | GC | 0.212 | 0.206/0.215 | 0.521 | 0.471 |
|  | GG | 0.055 | 0.058/0.054 | 0.418 | 0.518 |
| rs7062|rs56149204 |  |  |  |  |  |
|  | CG | 0.55 | 0.550/0.550 | 0.002 | 0.967 |
|  | TA | 0.271 | 0.270/0.271 | 0.001 | 0.978 |
|  | TG | 0.174 | 0.171/0.175 | 0.126 | 0.722 |
| rs1367448|rs1367447 |  |  |  |  |  |
|  | AC | 0.522 | 0.517/0.525 | 0.273 | 0.601 |
|  | GC | 0.255 | 0.255/0.254 | 0.008 | 0.929 |
|  | GT | 0.218 | 0.221/0.217 | 0.139 | 0.709 |
| rs59393705|rs2772297 |  |  |  |  |  |
|  | AC | 0.749 | 0.748/0.749 | 0.005 | 0.946 |
|  | CT | 0.211 | 0.212/0.210 | 0.022 | 0.881 |
|  | CC | 0.035 | 0.033/0.037 | 0.343 | 0.558 |
| rs2820503|rs17130583 |  |  |  |  |  |
|  | AC | 0.727 | 0.727/0.727 | 0.000 | 0.995 |
|  | GG | 0.231 | 0.225, 0.235 | 0.670 | 0.413 |
|  | GC | 0.036 | 0.040, 0.035 | 1.105 | 0.293 |
| rs4655788|rs12034030 |  |  |  |  |  |
|  | GT | 0.768 | 0.768, 0.769 | 0.002 | 0.961 |
|  | CC | 0.15 | 0.152, 0.149 | 0.102 | 0.750 |
|  | GC | 0.077 | 0.073, 0.079 | 0.680 | 0.410 |
| rs4655789|rs79249533 |  |  |  |  |  |
|  | TT | 0.54 | 0.541, 0.539 | 0.017 | 0.895 |
|  | GA | 0.319 | 0.320, 0.318 | 0.011 | 0.915 |
|  | TA | 0.135 | 0.132, 0.137 | 0.226 | 0.634 |

Supplemental Table S6. Results of single marker based linear regression analyses for OPG and sRANKL.

| CHR | SNP | BP | A1 | BETA_OPG | P_OPG | P_OPG_Con* | BETA_sRANKL | P_sRANKL | P_sRANKL_Con* |
| --- | --- | --- | --- | --- | --- | --- | --- | --- | --- |
| 1 | rs2772304 | 68636580 | A | 1.504 | 0.0277 | 0.0022 | -0.323 | 5.10×10^-6^ | 0.0541 |
| 1 | rs12725769 | 68637158 | C | 2.969 | 1.26×10^-5^ | 0.2576 | -0.435 | 7.24×10^-10^ | 0.5078 |
| 1 | rs59393705 | 68644066 | C | 6.514 | 4.23×10^-11^ | 0.0125 | -0.883 | < 1×10^-16^ | 0.0134 |
| 1 | rs2772297 | 68652084 | T | 7.081 | < 1×10^-16^ | 0.0002 | -0.977 | < 1×10^-16^ | 0.0003 |
| 1 | rs2026749 | 68653489 | G | 9.100 | < 1×10^-16^ | 9.19×10^-16^ | -1.261 | < 1×10^-16^ | 3.46×10^-11^ |
| 1 | rs2566752 | 68656697 | C | 11.870 | < 1×10^-16^ | - | -1.538 | < 1×10^-16^ | - |

* *P* values of linear regression analyses conditioned on rs2566752.

Supplemental Table S7. Single marker based linear regression for four quantitative traits using data from discovery stage.

| CHR | SNP | POS | BETA_LSBMD | *P*_LSBMD | BETA_FNBMD | *P*_FNBMD | BETA_OPG | *P*_OPG | BETA_sRANKL | *P*_sRANKL |
| --- | --- | --- | --- | --- | --- | --- | --- | --- | --- | --- |
| 1 | rs1033973 | 68566993 | 0.0031 | 0.4263 | 0.0003 | 0.9480 | 0.6063 | 0.6637 | -0.0493 | 0.7259 |
| 1 | rs11209214 | 68567299 | 0.0035 | 0.4575 | -0.0013 | 0.8095 | 0.0601 | 0.9715 | 0.0008 | 0.9964 |
| 1 | rs9436797 | 68567418 | 0.0060 | 0.2079 | 0.0023 | 0.6889 | 1.6890 | 0.3200 | -0.1954 | 0.2541 |
| 1 | rs11580462 | 68567441 | 0.0074 | 0.0187 | 0.0024 | 0.5157 | 1.5910 | 0.1547 | 0.0046 | 0.9677 |
| 1 | rs1033974 | 68567812 | 0.0076 | 0.0234 | 0.0033 | 0.4137 | 2.0470 | 0.0889 | -0.0036 | 0.9766 |
| 1 | rs1033975 | 68567848 | 0.0037 | 0.3205 | -0.0003 | 0.9461 | 1.2280 | 0.3569 | 0.1100 | 0.4132 |
| 1 | rs11805150 | 68568921 | -0.0003 | 0.9425 | 0.0020 | 0.7078 | -1.1980 | 0.4576 | 0.0605 | 0.7100 |
| 1 | rs6671127 | 68571220 | 0.0009 | 0.7639 | -0.0011 | 0.7539 | -0.6754 | 0.5176 | 0.0602 | 0.5675 |
| 1 | rs12738528 | 68576340 | 0.0002 | 0.9734 | -0.0032 | 0.6150 | -1.3510 | 0.4767 | 0.0688 | 0.7192 |
| 1 | rs187922 | 68576820 | 0.0000 | 0.9893 | -0.0003 | 0.9340 | 0.1814 | 0.8589 | 0.0138 | 0.8930 |
| 1 | rs76551413 | 68581841 | -0.0007 | 0.8907 | -0.0028 | 0.6272 | -0.0073 | 0.9966 | 0.0120 | 0.9451 |
| 1 | rs12741473 | 68581999 | 0.0002 | 0.9572 | 0.0007 | 0.8739 | 0.3994 | 0.7502 | -0.0214 | 0.8658 |
| 1 | rs2270 | 68585783 | 0.0024 | 0.6045 | 0.0028 | 0.6152 | 1.9150 | 0.2548 | -0.0998 | 0.5563 |
| 1 | rs2820502 | 68586404 | -0.0022 | 0.6761 | -0.0013 | 0.8286 | -1.5740 | 0.4004 | 0.0680 | 0.7189 |
| 1 | rs1046852 | 68591104 | 0.0031 | 0.2697 | -0.0012 | 0.7114 | -0.1034 | 0.9180 | -0.1499 | 0.1390 |
| 1 | rs7062 | 68591253 | 0.0032 | 0.2469 | -0.0018 | 0.5877 | 0.0921 | 0.9260 | -0.1402 | 0.1604 |
| 1 | rs56149204 | 68594560 | 0.0002 | 0.9574 | -0.0033 | 0.3615 | -0.1742 | 0.8747 | -0.0782 | 0.4828 |
| 1 | rs145687155 | 68601508 | -0.0023 | 0.6783 | -0.0011 | 0.8641 | -2.2560 | 0.2522 | -0.0711 | 0.7205 |
| 1 | rs2052958 | 68609355 | 0.0022 | 0.5357 | 0.0021 | 0.6133 | -0.8478 | 0.5076 | -0.1563 | 0.2257 |
| 1 | rs76918505 | 68623898 | -0.0058 | 0.2586 | -0.0056 | 0.3628 | -1.6520 | 0.3702 | 0.3335 | 0.0728 |
| 1 | rs1367448 | 68633924 | 0.0045 | 0.1033 | 0.0031 | 0.3535 | 0.9370 | 0.3446 | 0.0178 | 0.8585 |
| 1 | rs1367447 | 68634030 | 0.0047 | 0.1549 | 0.0037 | 0.3453 | 0.5946 | 0.6161 | 0.0226 | 0.8502 |
| 1 | rs2772304 | 68636580 | 0.0069 | 0.0325 | 0.0074 | 0.0507 | 1.1020 | 0.3368 | -0.2409 | 0.0373 |
| 1 | rs12725769 | 68637158 | 0.0146 | 5.99×10^-6^ | 0.0169 | 8.71×10^-6^ | 2.9990 | 0.0090 | -0.4064 | 0.0004 |
| 1 | rs59393705 | 68644066 | 0.0305 | < 1×10^-16^ | 0.0319 | < 1×10^-16^ | 5.9540 | 1.80×10^-7^ | -0.8884 | 9.49×10^-15^ |
| 1 | rs2772297 | 68652084 | 0.0343 | < 1×10^-16^ | 0.0368 | < 1×10^-16^ | 6.3990 | 8.79×10^-8^ | -1.0370 | < 1×10^-16^ |
| 1 | rs2026749 | 68653489 | 0.0467 | < 1×10^-16^ | 0.0487 | < 1×10^-16^ | 9.3510 | < 1×10^-16^ | -1.3530 | < 1×10^-16^ |
| 1 | rs2566752 | 68656697 | 0.0594 | < 1×10^-16^ | 0.0646 | < 1×10^-16^ | 13.1400 | < 1×10^-16^ | -1.6900 | < 1×10^-16^ |
| 1 | rs2116047 | 68661751 | 0.0207 | < 1×10^-16^ | 0.0178 | 6.82×10^-8^ | 5.1410 | 2.38×10^-7^ | -0.5349 | 9.72×10^-8^ |
| 1 | rs75437229 | 68662739 | 0.0122 | 0.0162 | 0.0075 | 0.2096 | 5.8080 | 0.0013 | -0.2033 | 0.2654 |
| 1 | rs7554551 | 68665023 | 0.0054 | 0.1131 | 0.0016 | 0.6837 | 0.8625 | 0.4743 | 0.0806 | 0.5074 |
| 1 | rs7511845 | 68665174 | 0.0024 | 0.4232 | -0.0001 | 0.9797 | -0.4675 | 0.6602 | 0.0616 | 0.5660 |
| 1 | rs2820503 | 68666724 | 0.0011 | 0.7350 | 0.0013 | 0.7216 | 0.4549 | 0.6829 | 0.1181 | 0.2929 |
| 1 | rs17130583 | 68674957 | 0.0014 | 0.6774 | 0.0025 | 0.5269 | 1.0770 | 0.3558 | 0.0516 | 0.6611 |
| 1 | rs4655788 | 68677550 | -0.0031 | 0.4214 | -0.0047 | 0.2998 | -0.1219 | 0.9290 | 0.1319 | 0.3386 |
| 1 | rs12034030 | 68677760 | 0.0022 | 0.5065 | -0.0028 | 0.4779 | 0.4555 | 0.7008 | -0.0231 | 0.8470 |
| 1 | rs76039723 | 68680366 | -0.0022 | 0.5972 | -0.0062 | 0.2107 | -0.1119 | 0.9401 | 0.0275 | 0.8546 |
| 1 | rs989842 | 68680929 | 0.0037 | 0.4756 | -0.0023 | 0.7118 | 0.0208 | 0.9910 | -0.1680 | 0.3682 |
| 1 | rs4655789 | 68694251 | 0.0010 | 0.7247 | -0.0022 | 0.5225 | -0.6716 | 0.5240 | -0.0506 | 0.6340 |
| 1 | rs79249533 | 68695408 | 0.0020 | 0.4636 | -0.0005 | 0.8787 | -0.9694 | 0.3305 | 0.0165 | 0.8696 |

Supplemental Table S8. RegulomeDB scores for 6 SNPs included in the validation stage.

| SNP | SCORE |
| --- | --- |
| rs2772304 | 5 |
| rs12725769 | 6 |
| rs59393705 | 6 |
| rs2772297 | 6 |
| rs2026749 | NA |
| rs2566752 | NA |
